# Decoding the ubiquitination-immunity axis in idiopathic pulmonary fibrosis: diagnostic insights and therapeutic implications

**DOI:** 10.1186/s12931-026-03612-7

**Published:** 2026-03-06

**Authors:** Wenjuan He, Chunmei Feng, Mengyuan Liu, Huan Qin, Yin Wu, Changwen Deng, Xiaoping Zhu

**Affiliations:** 1 Department of Pulmonary and Critical Care Medicine, Shanghai East Hospital, School of Medicine, Tongji University, Shanghai, China; 2 Shanghai Key Laboratory of Lung Inflammation and Injury, Shanghai, China; 3 Department of Pulmonary and Critical Care Medicine, Cangzhou Central Hospital, Cangzhou, Hebei Province China; 4 Shenzhen Ruipuxun Academy for Stem Cell & Regenerative Medicine, Shenzhen, 5181222 China

**Keywords:** Idiopathic pulmonary fibrosis, Ubiquitination, Diagnostic biomarker, Immune infiltration, Single-cell RNA sequencing, Ubiquitin D, Fibroblast activation

## Abstract

**Background:**

Idiopathic pulmonary fibrosis (IPF) is a chronic and progressive interstitial lung disease with limited therapeutic options. Emerging evidence suggests that ubiquitination-related genes (URGs) may contribute to IPF pathogenesis, although their diagnostic and immunological significance remains poorly understood.

**Methods:**

Three bulk RNA sequencing (RNA-seq) datasets (GSE110147, GSE53845, and GSE32537) and one single-cell RNA-seq dataset (GSE122960) were obtained from the Gene Expression Omnibus database. GSE110147 and GSE53845 were merged as the training cohort to construct a URG-based diagnostic model using least absolute shrinkage and selection operator (LASSO) regression, while GSE32537 and GSE122960 served as external validation cohorts. Immune cell infiltration was assessed using the CIBERSORT algorithm. A bleomycin-induced mouse model of pulmonary fibrosis was used to validate hub URG expression. The pro-fibrotic role of ubiquitin D (UBD) was evaluated in vitro using Cell Counting Kit-8 (CCK-8), wound healing, and transwell assays in mouse primary lung fibroblasts (PLFs). Transwell-based neutrophil migration assays were used to assess the impact of UBD expression in PLFs on neutrophil infiltration.

**Results:**

We developed a robust six-gene diagnostic signature with high predictive accuracy in both training and validation cohorts. Immune infiltration analysis revealed strong correlations between hub URGs and specific immune cell types. Among them, UBD showed the most significant upregulation in fibrotic lungs and was positively associated with neutrophil infiltration. Functional assays demonstrated that UBD silencing attenuated transforming growth factor β1 (TGFβ1)-induced fibroblast activation. Moreover, UBD knockdown significantly increased the expression levels of *Cxcl2* and *Cxcl3* in PLFs and promoted neutrophil migration.

**Conclusion:**

This study highlights the diagnostic and immunological relevance of URGs in IPF and identifies UBD as a key pro-fibrotic factor and neutrophil infiltration regulator, offering novel insights into IPF pathogenesis and potential therapeutic targets.

**Graphical abstract:**

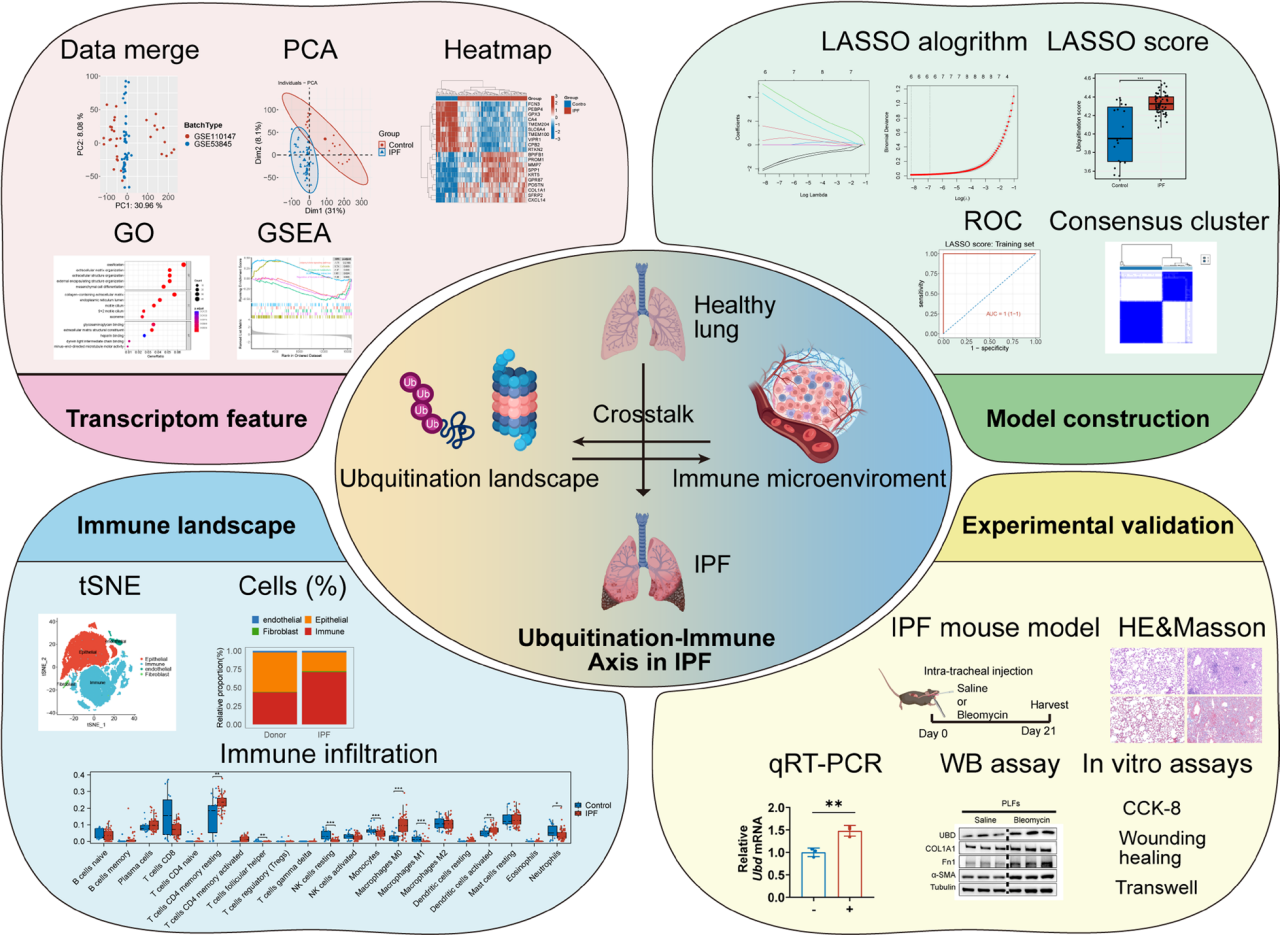

**Supplementary Information:**

The online version contains supplementary material available at 10.1186/s12931-026-03612-7.

## Background

Idiopathic pulmonary fibrosis (IPF) is a progressive and fatal form of interstitial lung disease of unknown etiology, characterized by relentless fibrotic remodeling of the lung parenchyma, aberrant extracellular matrix (ECM) deposition, and irreversible architectural distortion [1]. These pathological changes result in impaired lung compliance, respiratory insufficiency, and ultimately death [2]. IPF predominantly affects middle-aged and elderly individuals, with a rising global incidence and poor prognosis despite the use of antifibrotic agents such as pirfenidone and nintedanib [3–6]. The median survival after diagnosis remains approximately 2–3 years, underscoring the urgent need for more effective diagnostic and therapeutic strategies [5].

At the molecular level, IPF pathogenesis involves persistent alveolar epithelial injury, fibroblast activation, and excessive ECM accumulation [4, 7]. Among the diverse regulatory mechanisms implicated, ubiquitination—a critical post-translational modification—has recently emerged as a key modulator of fibrotic signaling [8]. This reversible enzymatic cascade, mediated by E1 activating enzymes, E2 conjugating enzymes, and E3 ligases, regulates the stability, localization, and activity of substrate proteins through the covalent attachment of ubiquitin [9]. Recent studies have identified specific ubiquitination-related molecules involved in IPF progression. For example, PKM2 promotes fibrogenesis by stabilizing TGFβ1 receptor I via reduced ubiquitination, thereby amplifying downstream signaling [10]. Conversely, RNF130 mitigates fibrosis by enhancing c-Myc ubiquitination and degradation, thereby inhibiting fibroblast-to-myofibroblast transition and aerobic glycolysis [11]. NEDD4 also contributes to fibrotic remodeling by regulating YY1 ubiquitination and modulating Table 1 transcription [12]. While these findings implicate ubiquitin signaling in IPF, a systematic investigation of ubiquitination-related genes (URGs) in this context remains lacking.

Immune dysregulation is another major contributor to IPF pathobiology [13]. Various immune cells—including macrophages and T lymphocytes—play complex and sometimes contradictory roles in fibrosis through the secretion of cytokines and growth factors [14]. However, the potential crosstalk between ubiquitination and immune infiltration within the fibrotic microenvironment remains largely unexplored, representing a critical gap in our mechanistic understanding.

In this study, we comprehensively profiled the ubiquitination landscape in IPF and constructed a six-gene diagnostic model that demonstrated strong predictive performance across both training and validation cohorts. We further dissected the immune microenvironment and identified significant associations between hub URGs and immune cell infiltration, suggesting an interplay between ubiquitin signaling and modulation of immune responses in the fibrotic niche. Finally, we validated the expression of these URGs in a bleomycin-induced mouse model and explored the pro-fibrotic function of UBD.

## Materials and methods

### Data collection and preprocessing

Three bulk RNA-seq datasets were downloaded from the GEO database: GSE110147 (11 control, 22 IPF), GSE53845 (8 control, 40 IPF), and GSE32537 (50 control, 119 IPF). To increase sample size and minimize bias, GSE110147 and GSE53845 were merged as the training cohort, with batch effects removed using the “ComBat” function from the sva R package. GSE32537 served as an independent external validation cohort. Additionally, the single-cell RNA-seq (scRNA-seq) dataset GSE122960 was retrieved to validate key findings.

### Identification of differentially expressed URGs

URGs were curated from published literature [15]. Differentially expressed genes (DEGs) were identified using the limma R package with thresholds of adjusted p value < 0.05 and |log₂FC| > 1. Overlapping genes between DEGs and URGs were defined as differentially expressed URGs (DEURGs) and subjected to further analysis.

### Functional annotation

Gene Ontology (GO) analysis was performed to explore biological processes (BP), cellular components (CC), and molecular functions (MF). KEGG pathway enrichment was conducted via Gene Set Enrichment Analysis (GSEA), using the clusterProfiler package with q-value < 0.05 and FDR < 0.25 as cutoffs [16]. Single-sample GSEA (ssGSEA) was used to evaluate URG-related pathway activities.

### Consensus clustering

Unsupervised clustering based on six hub URGs was performed using the ConsensusClusterPlus R package with 1,000 iterations to determine IPF subgroups [17].

### LASSO regression for model construction

Least Absolute Shrinkage and Selection Operator (LASSO) regression was applied via the glmnet package to construct a predictive model. The penalty parameter λ was optimized using 10-fold cross-validation. The ubiquitination score was calculated as: ubiquitination score =${\sum}_{i}^{n}(\mathrm{c}\mathrm{o}\mathrm{e}\mathrm{f}\mathrm{i}\times\mathrm{e}\mathrm{x}\mathrm{p}\mathrm{r}\mathrm{i})$, where *expri* is the expression of gene *i*, and *coefi* is its LASSO coefficient. Model performance was evaluated using ROC curve analysis (pROC package), and the area under curve (AUC) was calculated.

### scRNA-seq analysis

The GSE122960 scRNA-seq dataset was analyzed using Seurat (v4.0.3), following the standard workflow established by previous studies [18]. Genes expressed in fewer than 5 cells were removed; cells with fewer than 300 features were excluded. Louvain clustering was conducted using the top 15 principal components with a resolution of 0.006. Cell types were annotated using the following markers:

- Epithelial cells: EPCAM, KRT19, CLDN4
- Endothelial cells: CDH5, PECAM1, VWF
- Fibroblasts: LUM, FGF7, MME
- Immune cells: PTPRC

### Immune infiltration analysis

The relative abundance of 22 immune cell types between IPF and control samples in the training cohort was assessed using the CIBERSORT algorithm. Spearman correlation was used to determine associations between differentially infiltrating immune cells and hub URG expression.

### Animal model

Eight-week-old female C57BL/6 mice (Shanghai Model Organisms Center, Inc.) were maintained under specific-pathogen-free conditions. Pulmonary fibrosis was induced via intratracheal instillation of bleomycin sulfate (2 mg/kg in 50 µL saline). Controls received saline only. Lung tissues were harvested 21 days post-treatment. All procedures were approved by the Institutional Animal Care and Use Committee of Tongji University School of Medicine.

### Histological analysis

Lungs were fixed in 10% formalin, dehydrated, embedded in paraffin, sectioned (5 μm), and stained with H&E and Masson’s trichrome according to manufacturer protocols (Servicebio, Wuhan). H&E was used to assess alveolar damage and inflammation, and Masson’s to evaluate collagen deposition.

### RNA extraction and qRT-PCR

Total RNA was extracted using TRIzol (Invitrogen), and quantified using NanoDrop (Thermo Fisher). cDNA was synthesized using a high-fidelity reverse transcription kit (Takara). qRT-PCR was performed with SYBR Green Master Mix (Takara) on an Applied Biosystems 7300 system. Expression was normalized to β-actin (*Actb*) using the 2^^−ΔΔCt^ method. Primer sequences are listed in Table S1 [see Additional file 1].

### Western blotting

Protein was extracted using RIPA buffer with protease inhibitors. Lysates were centrifuged, and protein quantified via BCA assay. Samples (30 µg) were separated by SDS-PAGE, transferred to PVDF membranes, and blocked with 5% milk. Primary antibodies included UBD (Abclonal, A9005, 1:500), α-SMA (Affinity, AF1032, 1:1000), Collagen I (COL1A1) (abcam, ab138492, 1:3000), Fibronectin (Fn1) (Abclonal, A25907, 1:1000), alpha Tubulin (abcam, ab7291, 1:10000). After HRP-conjugated secondary antibody incubation, detection was done via ECL (Thermo Fisher), and band intensities quantified with ImageJ.

### Hydroxyproline assay

Lung hydroxyproline content was quantified using a commercial kit (A030-2; Jiancheng Bioengineering Institute) and normalized to tissue weight (µg/mg). Absorbance was read at 550 nm.

### Cell culture, treatment, and transfection

primary lung fibroblasts (PLFs) were isolated from C57BL/6 mouse lungs (untreated or post-bleomycin) and cultured in DMEM with 10% FBS, 1% penicillin/streptomycin. Fibroblast activation was induced by 48-hour treatment with TGFβ1 (10 ng/mL; Sigma). Two siRNAs targeting mouse *Ubd* and a control siRNA (GenePharma) were transfected into PLFs using Lipofectamine 2000 (Invitrogen) 24 h before TGFβ1 stimulation. The sequences of the *Ubd*-targeting siRNAs were:

- si-Ubd #1: 
- Sense: 5'-GGUUGUGAAUUGCAACGGATT-3'
- Antisense: 5'-UCCGUUGCAAUUCACAACCUU-3'
- si-Ubd #2:
- Sense: 5'-GAGAUGAUCGAGAGUGUGATT-3'
- Antisense: 5'-UCACACUCUCGAUCAUCUCTT-3'

### Neutrophil isolation

Murine neutrophils were isolated from bone marrow using a commercial neutrophil isolation kit (P9040; Solarbio) according to the manufacturer’s instructions with minor modifications. Briefly, bone marrow cells were flushed from the femurs and tibias of mice using RPMI-1640 medium supplemented with 10% FBS, as previously described [19]. Cells were centrifuged at 220 g for 5 min and resuspended in PBS. The cell suspension was layered sequentially onto density gradient solutions (Reagent A and Reagent C) and centrifuged at 800 g for 25 min. Neutrophils were collected from the interface between Reagent A and Reagent C, washed with PBS, and centrifuged at 250 g for 10 min. Residual red blood cells were removed using red blood cell lysis buffer, followed by washing with PBS. Purified neutrophils were finally resuspended in RPMI-1640 medium for subsequent experiments.

### CCK-8 proliferation assay

Cells (3000/well) were seeded into 96-well plates, treated, then incubated with 10 µL CCK-8 reagent (MedChemExpress) for 2 h at 37 °C. Absorbance at 450 nm was measured to evaluate viability.

### Migration assays

#### PLF migration

For wound healing, a linear scratch was made in confluent cells, and migration was monitored in serum-free medium. For transwell assays, 3 × 10⁴ cells were seeded in the upper chamber; 10% FBS-containing medium was added to the lower chamber. After 24 h, migrated cells were stained with crystal violet and imaged.

#### Neutrophil migration

Neutrophil migration was assessed using transwell inserts with a 3-µm pore size (Corning). Briefly, 1 × 10⁵ neutrophils suspended in serum-free RPMI-1640 medium were seeded into the upper chamber. For the conditioned medium-based assay, the lower chamber was filled with culture supernatants collected from control or UBD-knockdown PLFs. For the cell-based co-culture assay, control or UBD-knockdown PLFs were seeded in the lower chamber and allowed to condition the medium prior to neutrophil seeding. To facilitate neutrophil migration, 50 nM N-formyl-Met-Leu-Phe (fMLP) (HY-P0224; MedChemExpress) was added to the lower chamber. Cells were incubated at 37 °C with 5% CO_2_ for 4 h. After incubation, neutrophils that had migrated into the lower chamber were collected by transferring the medium to a clean 24-well plate, imaged under an optical microscope, and quantified by cell counting.

### Immunofluorescence

PLFs on coverslips were fixed, permeabilized, and blocked with BSA. Cells were incubated overnight at 4 °C with primary antibodies against UBD (Abclonal, A9005, 1:100) and α-SMA (Affinity, AF1032, 1:200), followed by FITC-labeled secondary antibody. Nuclei were stained with DAPI, and images captured using a Leica confocal microscope.

### Statistical analysis

Statistical analyses were performed using GraphPad Prism 8 and R v4.1.1. Data are presented as mean ± SD. For two-group comparisons, a two-tailed unpaired Student’s t-test was used. For multiple groups, one-way ANOVA with Tukey’s test was applied. Nonparametric data were analyzed using the Kruskal-Wallis or Wilcoxon tests. Correlations were assessed with Spearman’s coefficient. A p value < 0.05 was considered statistically significant.

## Results

### Identification of DEGs in IPF

**Fig. 1:**
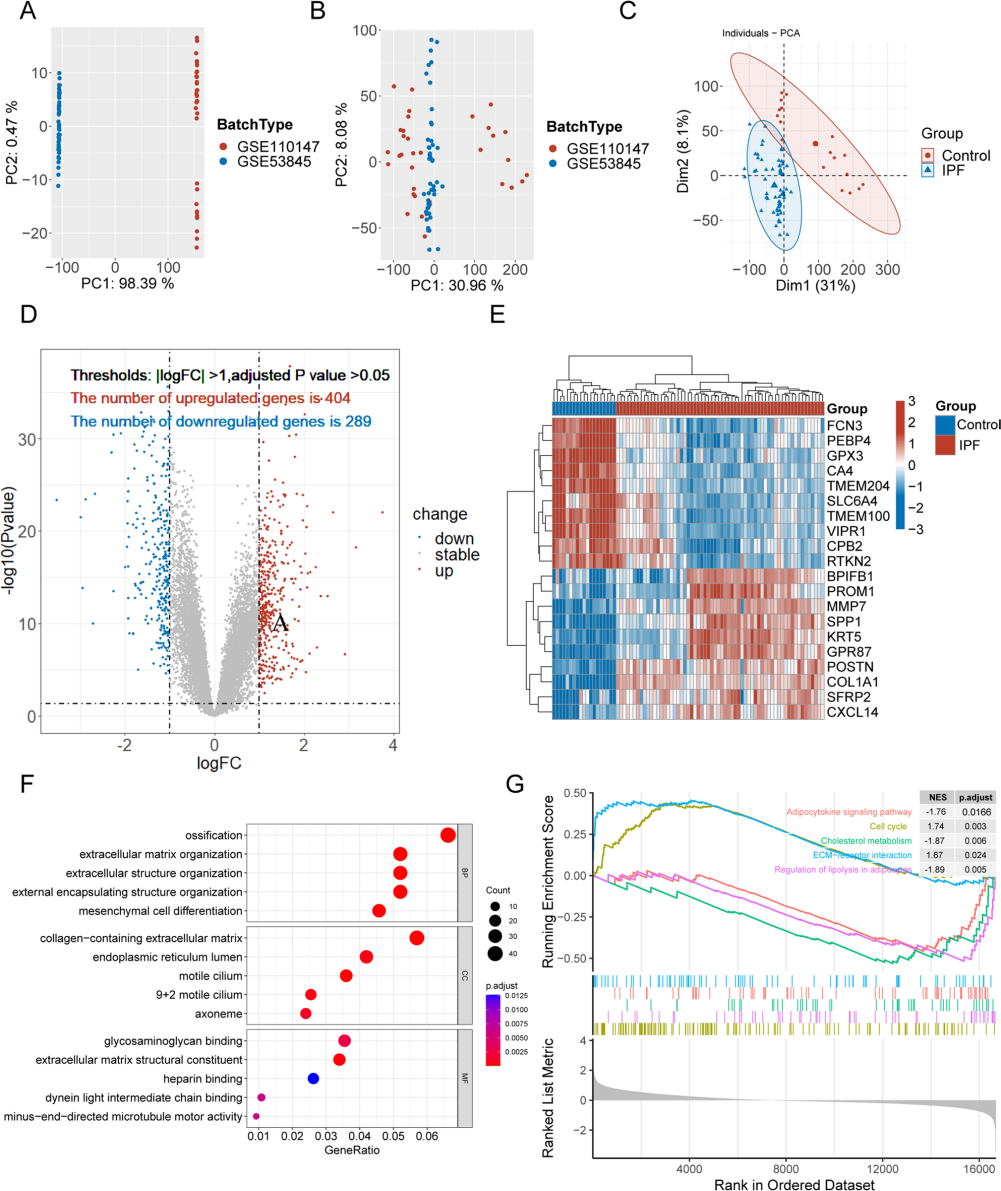
Identification of Differentially Expressed Genes (DEGs) in IPF. **A** Principal component analysis (PCA) of samples from two GEO datasets before batch effect correction. **B** PCA after batch effect correction using the ComBat algorithm. **C** PCA of the merged training cohort. **D** Volcano plot showing DEGs between IPF and control samples. Red and blue dots indicate upregulated and downregulated genes, respectively. **E** Heatmap of the top 10 upregulated and downregulated DEGs. **F** GO enrichment analysis highlighting biological processes associated with DEGs.**G** GSEA identifying enriched KEGG pathways in IPF versus control samples

To increase the sample size of the training set, datasets GSE110147 and GSE53845 were merged, and batch effects were effectively eliminated (Fig. 1A-B). The integrated training cohort consisted of 62 IPF lung tissue samples and 19 healthy controls. Principal component analysis (PCA) revealed a clear separation between IPF and control groups, indicating reliable sample stratification (Fig. 1C). A total of 693 DEGs were identified (Fig. 1D), with the top 10 upregulated and downregulated genes visualized in a heatmap (Fig. 1E). GO enrichment analysis showed that these DEGs were mainly involved in extracellular matrix organization, mesenchymal cell differentiation, and collagen-containing extracellular matrix components (Fig. 1F), consistent with established fibrotic mechanisms. GSEA further highlighted pathways such as adipocytokine signaling, cell cycle regulation, and cholesterol metabolism as potentially contributing to IPF pathogenesis (Fig. 1G).

### Construction of a URG-based diagnostic model for IPF

**Fig. 2:**
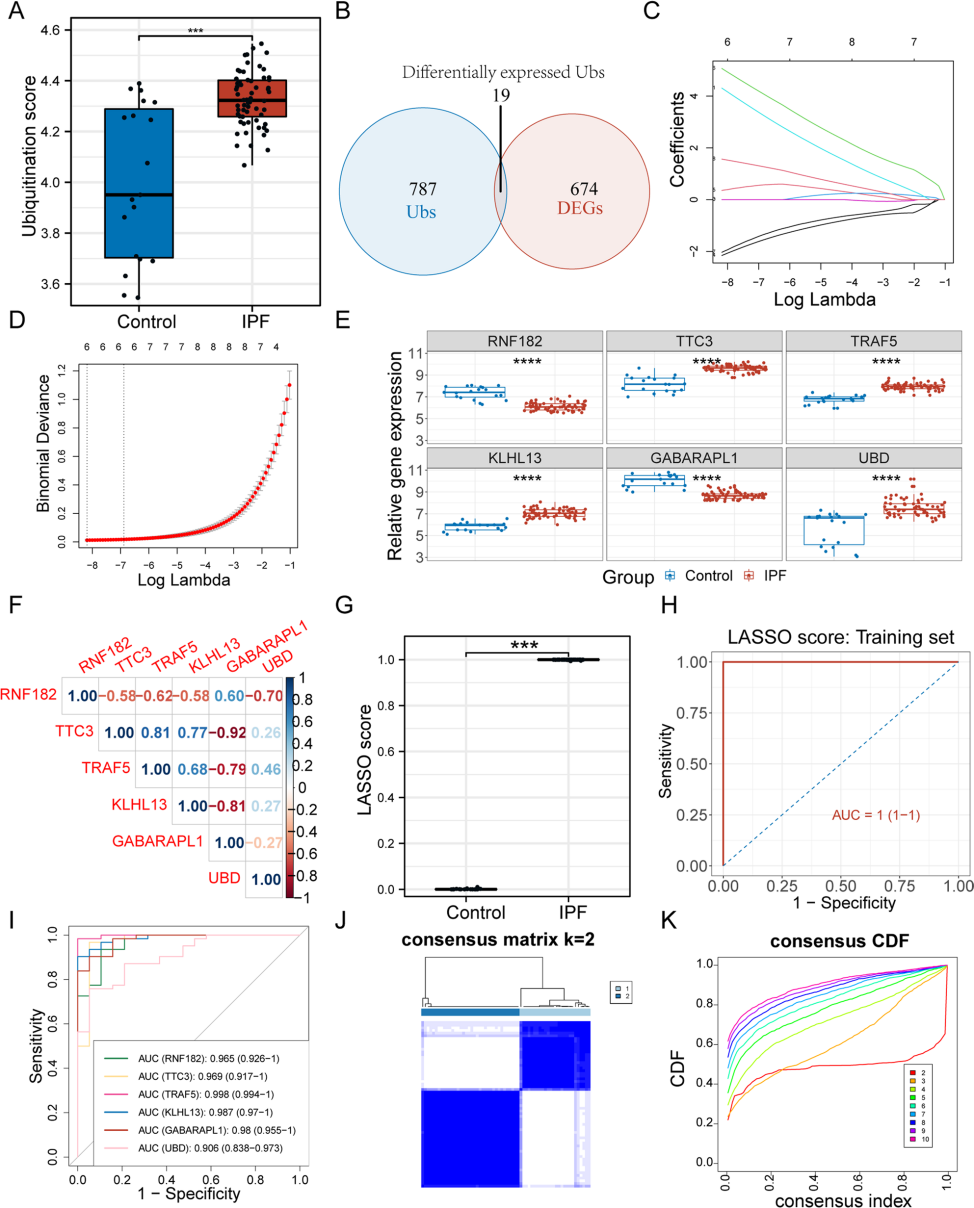
Construction of a URG-Based Diagnostic Model for IPF. **A** Ubiquitination scores in control and IPF samples calculated via ssGSEA. **B** Venn diagram showing the overlap between URGs and DEGs. **C**-**D** LASSO regression for feature selection and parameter tuning.**E** Boxplots showing differential expression of the six hub URGs between IPF and control samples. **F** Spearman correlation matrix among the six hub URGs. **G** Comparison of LASSO scores between IPF and control samples. **H** ROC curve evaluating diagnostic performance of the LASSO score.(I) Individual ROC curves for each of the six hub URGs. **J** Consensus clustering matrix of IPF samples based on the six hub URGs.

To explore the diagnostic potential of URGs in IPF, we used the ssGSEA algorithm to quantify the expression profiles of 806 URGs. The analysis revealed a significant ubiquitin imbalance between IPF and control samples (Fig. 2A). Among these, 19 URGs were differentially expressed (Fig. 2B).

LASSO regression was subsequently applied for feature selection and dimensionality reduction (Fig. 2C-D), resulting in the identification of six hub URGs: *RNF182*, *TTC3*, *TRAF5*, *KLHL13*, and *UBD* were upregulated, while *GABARAPL1* was downregulated in IPF (Fig. 2E). These hub genes exhibited strong intercorrelations (Fig. 2F), indicating potential co-regulatory relationships. A diagnostic model based on these six genes (LASSO score) demonstrated high discriminatory power (Fig. 2G-H), and each gene independently showed good diagnostic performance (Fig. 2I). Consensus clustering based on these six URGs revealed two distinct molecular subtypes among IPF patients, indicating potential heterogeneity within the disease (Fig. 2J-K). These findings support the establishment of a robust and clinically relevant URG-based diagnostic model.

### Validation of the URG-based diagnostic model

**Fig. 3:**
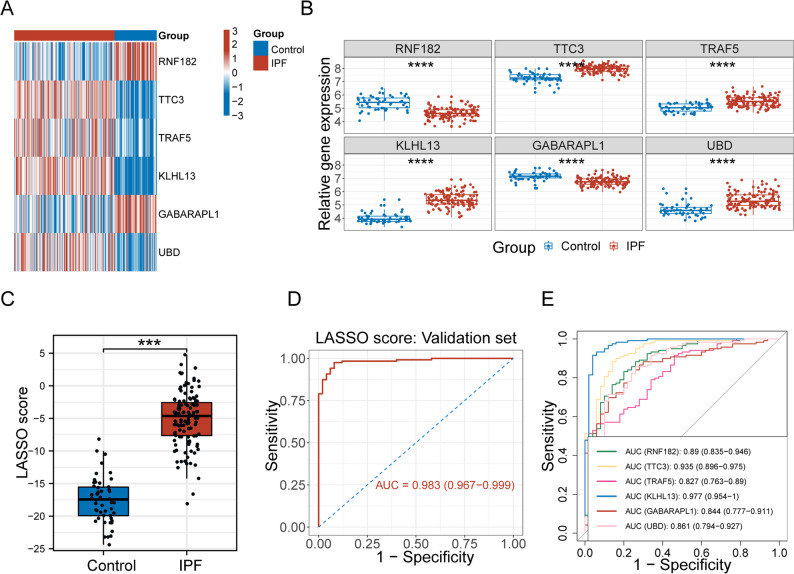
Validation of the URG-Based Diagnostic Model in the GSE32537 Cohort. **A** Heatmap of the six hub URGs in IPF and control samples. **B** Boxplots showing differential gene expression. **C** Comparison of LASSO scores between groups. **D** ROC curve of the LASSO score in the validation cohort. **E** Individual ROC curves of the six hub URGs. *p < 0.05, **p < 0.01, ***p < 0.001, ****p < 0.0001, *ns* not significant

To assess the reliability of the URG-based diagnostic model, we validated its performance in an independent RNA-seq dataset. The expression patterns of the six hub genes were consistent with those in the training cohort (Fig. 3A-B), and the calculated LASSO scores remained significantly elevated in IPF samples (Fig. 3C). The model exhibited excellent diagnostic performance in the validation dataset, with an AUC of 0.983 (Fig. 3D). Each hub URG also showed strong individual predictive power, with AUC values ranging from 0.827 to 0.977 (Fig. 3E).

**Fig. 4:**
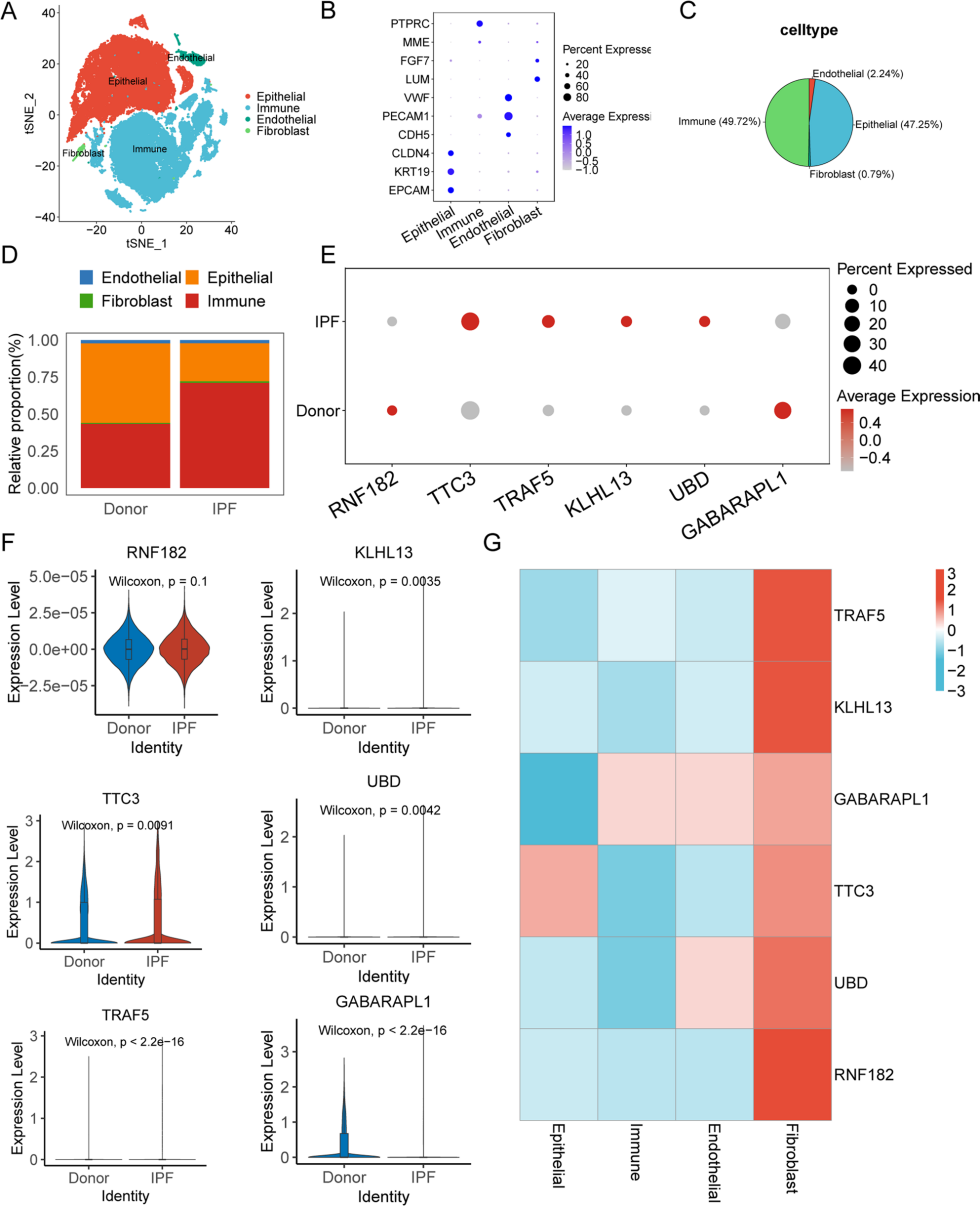
Validation of Hub URG Expression in Single-Cell RNA-seq Data. **A** tSNE plot showing clustering of four major lung cell types. **B** Expression of canonical marker genes for epithelial, endothelial, fibroblast, and immune cells. **C** Cell type proportions across all samples. **D** Stacked bar plot comparing cell-type composition between control and IPF samples. **E** Dot plot showing the average expression levels of the six hub URGs across all cell types in control and IPF samples. **F** Violin plots showing the average expression levels of the six hub URGs across all cell types in control and IPF samples. **G** Heatmap displaying expression trends of the six hub URGs across cell types

To further verify the expression and cellular specificity of the hub URGs, we analyzed a publicly available scRNA-seq dataset from IPF and healthy lung tissues. Cells were clustered into four main types: epithelial, immune, fibroblast, and endothelial cells (Fig. 4A-B). Immune and epithelial cells predominated in lung tissue, accounting for 49.72% and 47.25% of all cells, respectively (Fig. 4C). In IPF samples, the proportions of different cell types shifted markedly, with decreased epithelial cells and increased immune and fibroblast populations (Fig. 4D). Most hub URGs displayed expression trends in scRNA-seq data consistent with those observed in the bulk dataset, with the exception of *RNF182* (Fig. 4E-F). Notably, all six hub URGs showed specific enrichment in fibroblasts (Fig. 4G), suggesting a fibroblast-specific role in IPF pathogenesis. These results underscore the robustness and cross-platform applicability of our diagnostic model.

### Relationship Between Hub URGs and the Immune Landscape in IPF

**Fig. 5:**
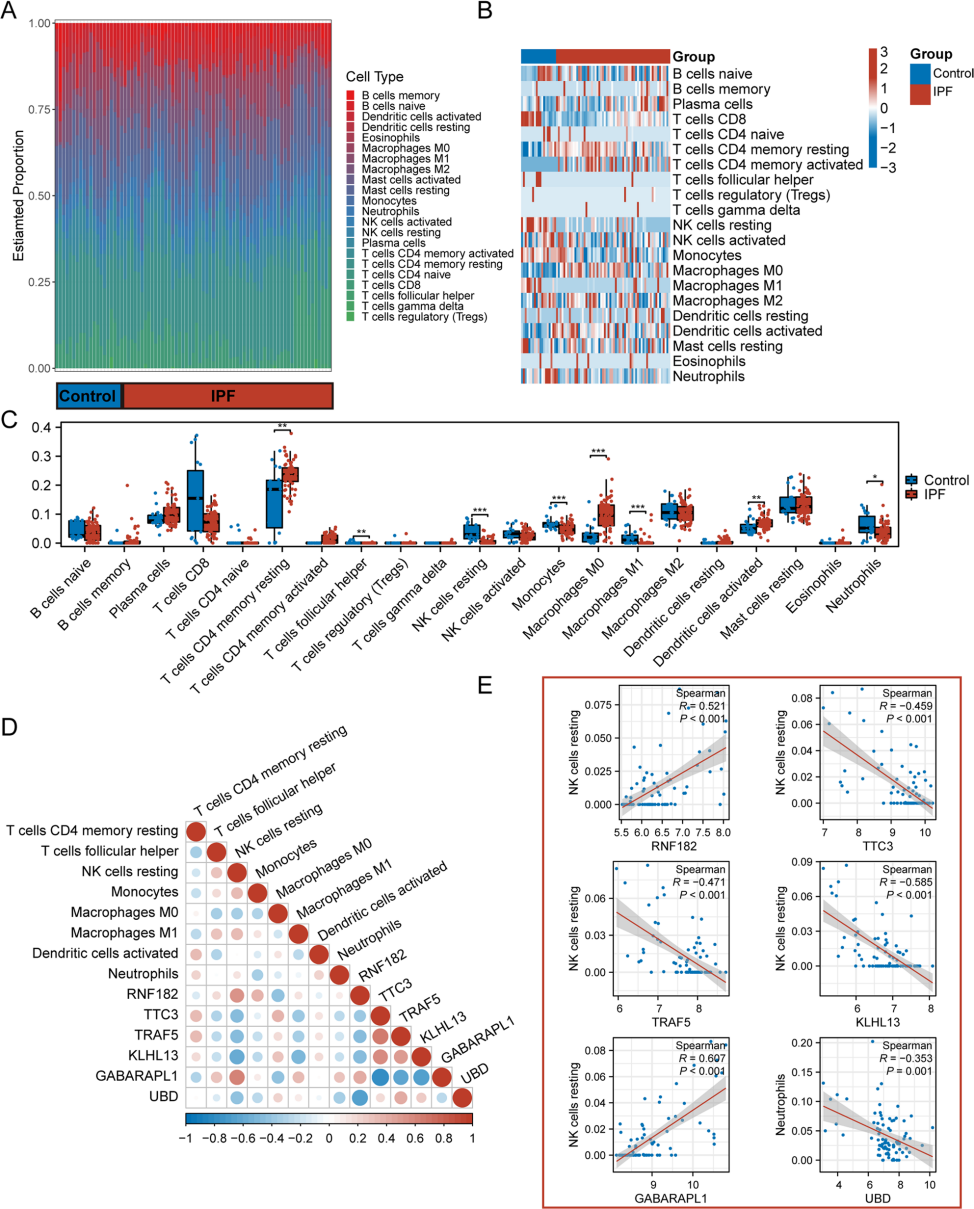
Association Between Hub URGs and Immune Cell Infiltration. **A** Bar plot of relative proportions of 22 immune cell types per sample. **B** Heatmap showing immune infiltration levels across samples. **C** Boxplots comparing immune cell infiltration between IPF and control groups. **D** Spearman correlations between the six hub URGs and eight differentially infiltrated immune cell types. **E** Scatter plots illustrating key correlations between hub URGs and specific immune cells. *p < 0.05, **p < 0.01, ***p < 0.001, ****p < 0.0001, ns: not significant

Considering the central role of immune dysregulation in IPF, we analyzed immune cell infiltration patterns. A bar plot showed the relative abundance of 22 immune cell types per sample (Fig. 5A), and a heatmap displayed infiltration differences between IPF and control groups (Fig. 5B). Eight immune cell subsets exhibited significant changes: T cells CD4 memory resting, M0 macrophages, and activated dendritic cells were increased in IPF, while follicular helper T cells, resting NK cells, monocytes, M1 macrophages, and neutrophils were decreased (Fig. 5C).

We then evaluated correlations between the six hub URGs and the eight differentially infiltrated immune cell populations (Fig. 5D). *RNF182*, *TTC3*, *TRAF5*, *KLHL13*, and *GABARAPL1* were most correlated with resting NK cells, while *UBD* showed a strong negative correlation with neutrophils (Fig. 5E). These findings suggest that dysregulated ubiquitin signaling may contribute to immune alterations in IPF and participate in shaping the fibrotic immune microenvironment.

### Validation of the Hub URGs in the bleomycin-induced IPF mouse model

**Fig. 6:**
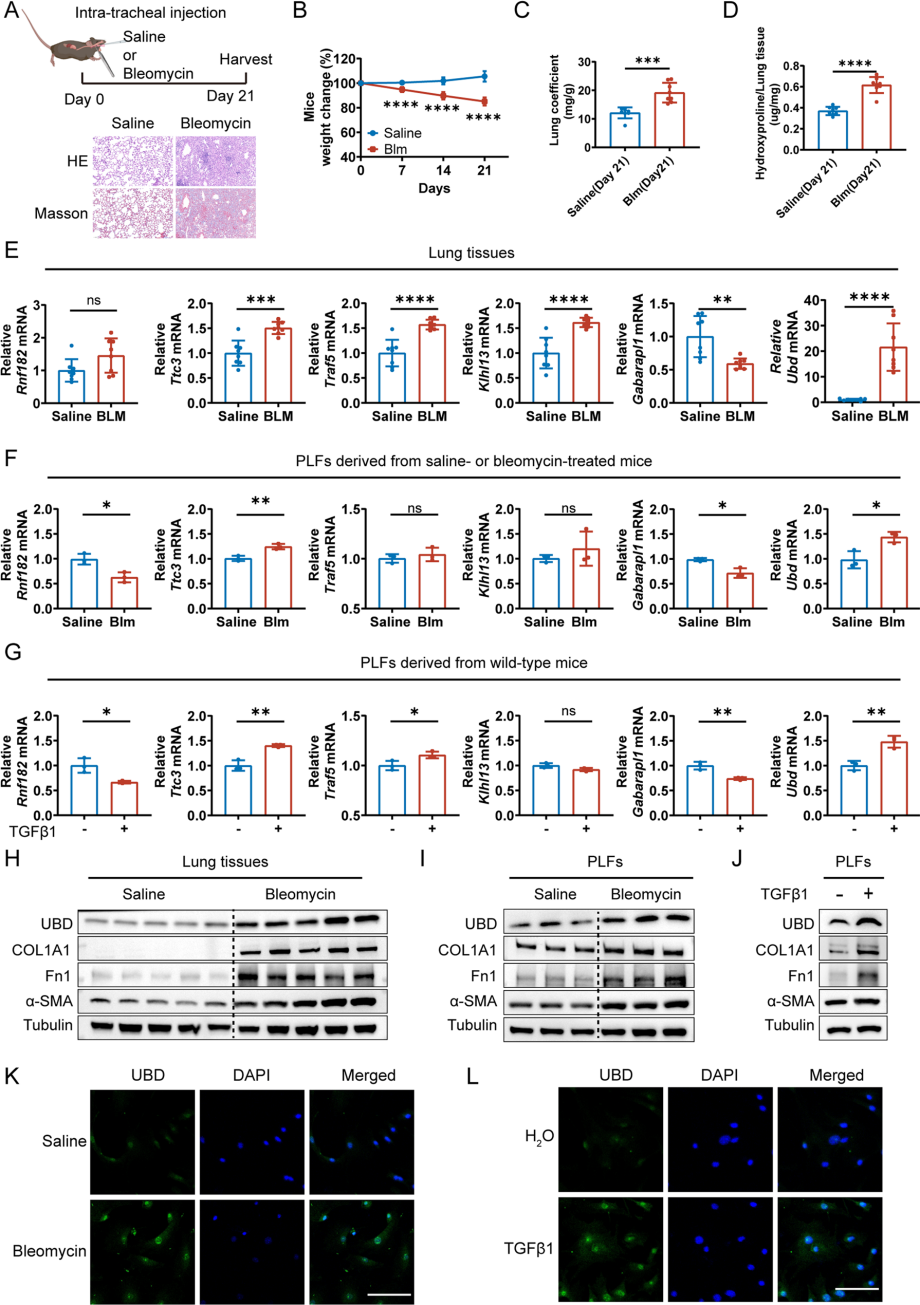
Identification of Ubd as a Key Pro-Fibrotic Gene. **A** H&E and Masson staining confirming successful induction of pulmonary fibrosis in mice. **B** Body weight changes over time in saline- and BLM-treated mice (*n* = 8 per group). **C** Lung coefficient (wet lung weight/body weight) in each group (*n* = 8).D Hydroxyproline content in lung tissue (*n* = 8). **E** mRNA expression of the six hub URGs in lung tissue (*n* = 8). **F** mRNA expression of hub URGs in primary lung fibroblasts (PLFs) from saline- or BLM-treated mice (*n* = 3). **G** Gene expression changes in PLFs after TGFβ1 stimulation (10 ng/mL, 24 h). **H** Western blot of UBD protein in lung tissues from saline- and BLM-treated mice (*n* = 5). **I** Western blot of UBD protein in PLFs from saline- or BLM-treated mice (*n* = 3). **J** Western blot showing UBD upregulation in PLFs after TGFβ1 treatment. **K** Immunofluorescence showing increased UBD in PLFs from BLM-treated mice (*n* = 3). Scale bar = 100 μm. **L** Immunofluorescence showing UBD induction in TGFβ1-treated PLFs. Scale bar = 100 μm.All PLFs were derived from untreated wild-type mice unless otherwise specified. *p < 0.05, **p < 0.01, ***p < 0.001, ****p < 0.0001, ns: not significant

To validate hub URG expression in vivo, we established a bleomycin (BLM)-induced pulmonary fibrosis model. Mice treated with BLM exhibited disrupted alveolar structures, thickened interstitial septa, and increased collagen deposition compared to saline-treated controls (Fig. 6A). BLM treatment also resulted in significant reductions in body weight and lung coefficient, along with elevated lung hydroxyproline content (Fig. 6B-D). The mRNA levels of most hub URGs in fibrotic lung tissue were consistent with the training dataset, with *Ubd* showing the most prominent upregulation (Fig. 6E).

Given the predominant expression of these hub URGs in fibroblasts, we further assessed their expression in PLFs. The expression profiles of *Ttc3*, *Gabarapl1*, and *Ubd* in PLFs—either isolated from BLM-treated mice or stimulated with TGFβ1—aligned with trends from the bulk data (Fig. 6F-G). Since *Ubd* exhibited the most pronounced and consistent upregulation in both lung tissue and PLFs, we evaluated its protein expression. Western blot analysis confirmed significantly elevated UBD levels in both fibrotic lungs and PLFs upon BLM or TGFβ1 treatment (Fig. 6H-J). Immunofluorescence staining also demonstrated enhanced UBD expression in PLFs after fibrosis induction (Fig. 6K-L). These results support the translational relevance of our bioinformatic findings and highlight UBD as a potential pro-fibrotic effector gene.

### UBD is functionally required for TGFβ1-induced lung fibroblast activation

**Fig. 7:**
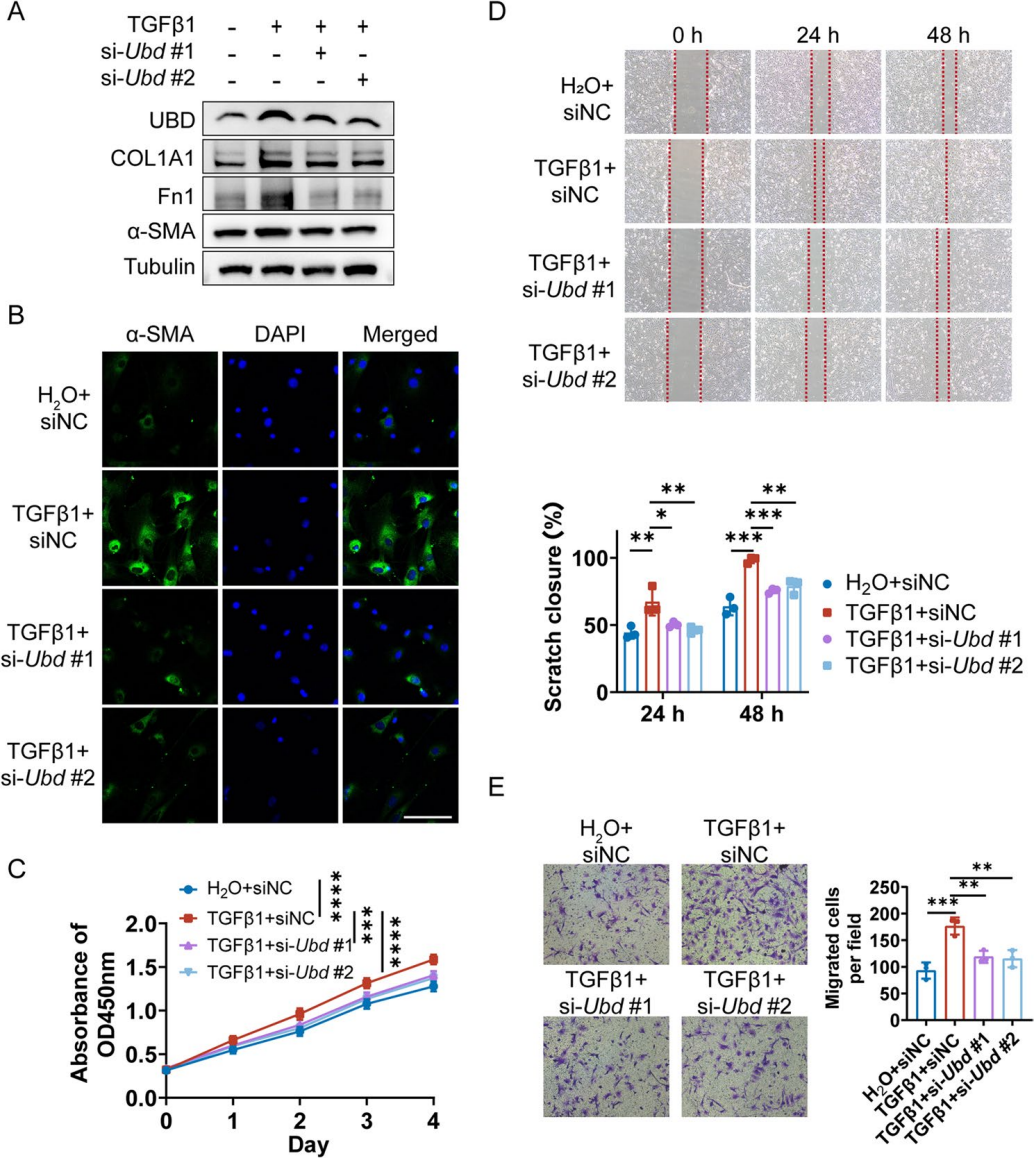
UBD Is Functionally Required for TGFβ1-Induced Fibroblast Activation. **A** Western blot showing reduced expression of Fn1, COL1A1, and α-SMA in TGFβ1-treated PLFs after Ubd knockdown. **B** Immunofluorescence showing decreased α-SMA signal following Ubd silencing in TGFβ1-treated PLFs. Scale bar = 100 μm. **C** CCK-8 assay showing reduced proliferation in Ubd-knockdown PLFs. **D** Wound healing assay indicating impaired migration upon Ubd silencing. **E** Transwell assay showing reduced migration in Ubd-knockdown PLFs. All PLFs were isolated from untreated wild-type mice unless otherwise indicated. *p < 0.05, **p < 0.01, ***p < 0.001, ****p < 0.0001, ns: not significant

To explore the functional role of UBD in fibroblast activation, we performed siRNA-mediated knockdown of *Ubd* in PLFs. Silencing UBD markedly reduced the TGFβ1-induced expression of Fn1, COL1A1, and α-SMA (Fig. 7A). Immunofluorescence staining confirmed a substantial decrease in α-SMA signal intensity following *Ubd* knockdown in TGFβ1-treated PLFs (Fig. 7B). Moreover, functional assays demonstrated that *Ubd* silencing significantly suppressed the proliferation and migration of PLFs in response to TGFβ1, as shown by CCK-8, wound healing, and transwell assays (Fig. 7C-E). Together, these findings establish UBD as a critical regulator of fibroblast activation and fibrogenic behavior, underscoring its potential as a therapeutic target in pulmonary fibrosis.

### UBD knockdown in PLFs facilitates neutrophil migration

Based on our immune infiltration analysis, UBD expression showed the strongest negative correlation with neutrophil infiltration in IPF samples. We therefore investigated whether UBD expression in fibroblasts modulates neutrophil migration.

**Fig. 8:**
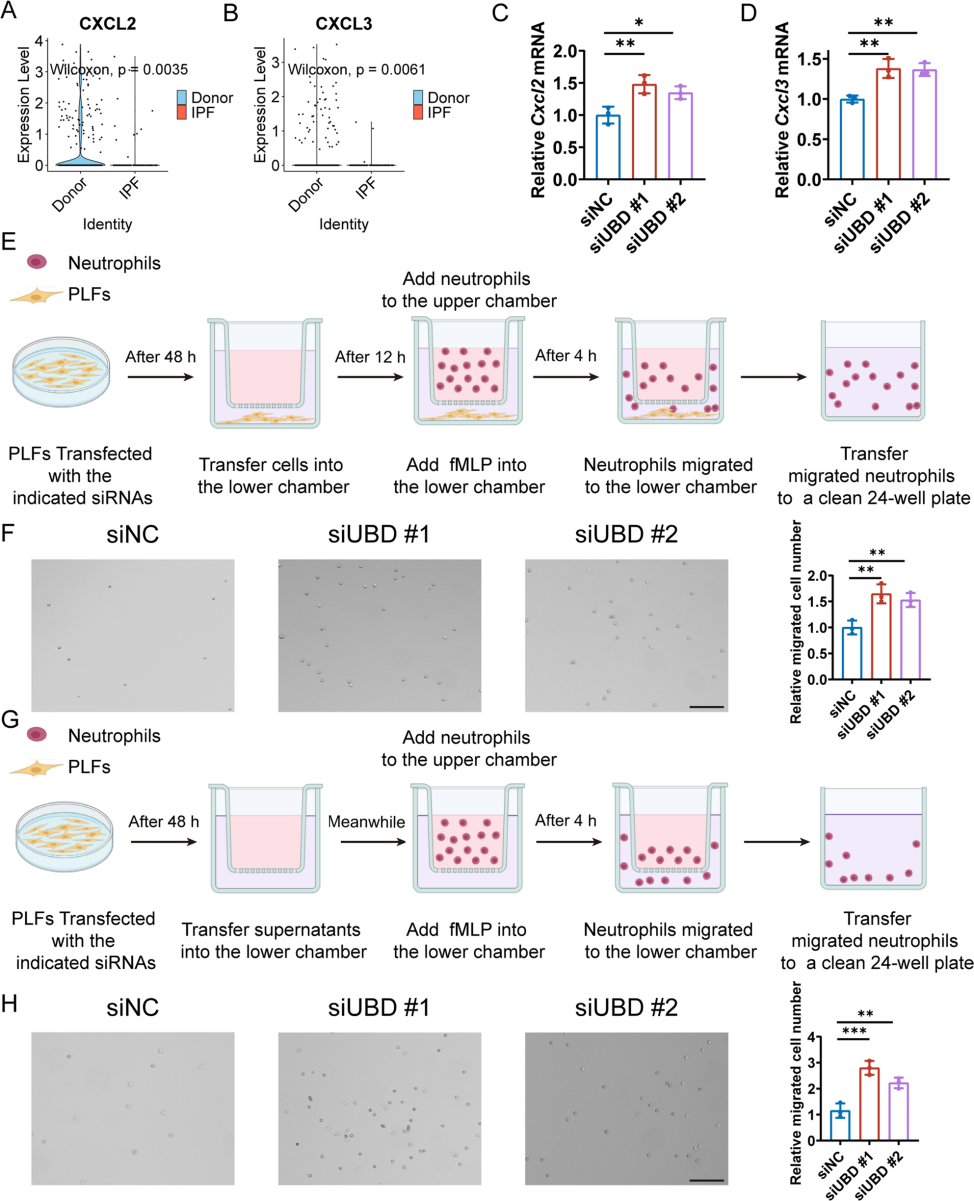
UBD knockdown in fibroblasts facilitates neutrophil migration.**A**-**B** Differential expression of *CXCL2* (**A**) and *CXCL3* (**B**) in fibroblasts from human IPF and control lungs in the scRNA-seq dataset GSE122960.**C**-**D** RT-qPCR analysis showing increased expression of *Cxcl2* (**C**) and *Cxcl3* (**D**) in mouse PLFs following *Ubd* knockdown.**E** Schematic illustration of the cell-based transwell co-culture assay. PLFs were transfected with the indicated siRNAs for 48 h and then seeded in the lower chamber. After 12 h of culture, 50 nM N-formyl-Met-Leu-Phe (fMLP) was added to the lower chamber, and neutrophils were placed in the upper chamber. After 4 h, medium from the lower chamber containing migrated neutrophils was collected, transferred to a clean 24-well plate, and neutrophils were imaged and counted under an optical microscope.**F** Representative images and quantification of neutrophil migration toward control or *Ubd*-knockdown PLFs in the cell-based transwell assay. Scale bar = 50 μm.**G** Schematic illustration of the conditioned medium-based transwell migration assay. PLFs were transfected with the indicated siRNAs for 48 h, after which culture supernatants were collected and transferred to the lower chamber. 50 nM fMLP was added, and neutrophils were placed in the upper chamber. After 4 h, medium from the lower chamber was collected, transferred to a clean 24-well plate, and migrated neutrophils were imaged and counted.**H** Representative images and quantification of neutrophil migration induced by conditioned media from control or *Ubd*-knockdown PLFs. Scale bar = 50 μm. All PLFs were isolated from untreated wild-type mice unless otherwise indicated. *p < 0.05, **p < 0.01, ***p < 0.001, ****p < 0.0001, ns: not significant

First, fibroblast subsets were extracted from the human scRNA-seq dataset GSE122960, and the expression of well-established neutrophil chemotactic ligands was systematically analyzed. These ligands included *CXCL1*, *CXCL2*, *CXCL3*, *CXCL5*, *CXCL6*, *PPBP* (the coding gene of CXCL7), *CXCL8*, *CCL3*, and *CCL4*, which are known to regulate neutrophil recruitment in human tissues. Differential expression analysis revealed that *CXCL2* and *CXCL3* were downregulated in fibroblasts from IPF patients compared to donor controls (Fig. 8A-B), whereas the other chemokines showed no significant differences (Figure S1[see Additional file 2]).

To functionally validate these observations, *Ubd* was knocked down in PLFs. Consistent with the potential regulatory role suggested by the human single-cell data, *Ubd* knockdown significantly increased the expression of *Cxcl2* and *Cxcl3* (Fig. 8C-D).

Finally, we assessed whether UBD deficiency in PLFs affects neutrophil migratory capacity using transwell assays. In the cell-based co-culture system, control or *Ubd*-knockdown PLFs were seeded in the lower chamber, with neutrophils added to the upper chamber (Fig. 8E). Neutrophil migration toward *Ubd*-deficient PLFs was significantly increased compared with controls (Fig. 8F). Similarly, in the conditioned medium-based assay, supernatants collected from *Ubd*-knockdown PLFs markedly enhanced neutrophil migration relative to control supernatants (Fig. 8G-H).

Collectively, these results demonstrate that suppression of UBD in fibroblasts promotes neutrophil migration, likely through upregulation of key neutrophil chemotactic factors such as CXCL2 and CXCL3, highlighting a fibroblast-mediated mechanism by which UBD modulates the fibrotic immune microenvironment in IPF.

## Discussion

IPF is a chronic interstitial lung disease characterized by progressive fibrosis and poor clinical outcomes [20]. Despite advances in imaging and histopathology, distinguishing IPF from other interstitial lung diseases remains challenging due to overlapping features and the absence of specific biomarkers [4]. The complex and largely unresolved pathogenesis further complicates early diagnosis and individualized treatment [21]. Therefore, identifying reliable molecular markers and uncovering potential regulatory mechanisms is essential to improve diagnostic accuracy and inform therapeutic strategies. Recent advances in bioinformatics and systems biology have provided powerful tools to investigate intricate genetic alterations driving disease progression across diverse cellular contexts [22].

In this study, we observed significantly altered transcriptomic profiles between IPF patients and healthy controls. GO analysis revealed that the DEGs were primarily enriched in ECM-related processes, such as ECM organization, collagen-containing matrix, and structural ECM components—hallmarks of IPF pathology [23, 24]. In addition, GSEA indicated that lipid and cholesterol metabolic pathways may be involved in IPF progression. Supporting this, lipidomic studies have shown that metabolites such as fatty acids, cholesterol, arachidonic acid derivatives, and phospholipids can promote fibrosis by inducing endoplasmic reticulum stress, apoptosis, and pro-fibrotic signaling [25]. These findings suggest that targeting lipid metabolism may represent a potential therapeutic avenue for IPF.

Ubiquitination is a conserved post-translational modification critical for maintaining protein homeostasis and regulating intracellular signaling [26]. Although dysregulated ubiquitination has been implicated in IPF pathogenesis [8], current evidence remains fragmented. In this study, we identified global alterations in ubiquitin activity using ssGSEA analysis of 806 URGs, indicating a ubiquitination imbalance in IPF. Among these, 19 URGs were differentially expressed between IPF and control samples. LASSO regression identified six hub URGs with strong diagnostic potential. A composite index based on these six genes exhibited excellent diagnostic performance in both the training and validation cohorts, highlighting their promise as novel IPF biomarkers. Moreover, consensus clustering revealed two molecular subtypes of IPF, suggesting the existence of underlying heterogeneity and the potential for subtype-specific treatment strategies.

Functionally, RNF182, a brain-enriched E3 ligase, has been linked to Alzheimer and hepatocellular carcinoma through degradation of ATP6V0C and p65, respectively [27, 28]. TRAF5 has been shown to inhibit cardiac hypertrophy via negative regulation of the MEK-ERK1/2 pathway [29], but also contributes to liver fibrosis by enhancing glycolysis in hepatic stellate cells [30]. TTC3 was reported to alleviate liver fibrosis through regulation of KIF18A and inhibition of the AKT/mTOR pathway [31]. Zhao et al. identified KLHL13 as an IPF biomarker, consistent with our findings [32]. GABARAPL1, a marker of glycophagy, suppresses hypoxia-induced pyroptosis in endothelial cells [33, 34]. UBD (also known as FAT10), previously shown to promote liver fibrosis via SIRT1 modulation [35], was identified in our study as the most upregulated hub gene in fibrotic lung tissues and fibroblasts.

The expression trends of most hub URGs were consistent across the training set, scRNA-seq dataset, and the BLM-induced IPF mouse model. Notably, these URGs were predominantly enriched in fibroblasts, a cell type central to IPF progression [36]. Among them, *Ttc3*, *Gabarapl1*, and *Ubd* showed consistent upregulation in PLFs from BLM-treated mice and in TGFβ1-stimulated PLFs, suggesting a role in fibroblast activation. Functional experiments demonstrated that *Ubd* silencing attenuated TGFβ1-induced fibroblast activation, highlighting its contribution to pro-fibrotic processes.

Immune dysregulation has increasingly been recognized as a key driver of IPF progression [14]. Our scRNA-seq and immune infiltration analyses revealed altered immune cell populations in IPF lung tissues. Interestingly, five hub genes were most associated with resting NK cells, while *UBD* showed a strong negative correlation with neutrophils. Indeed, functional experiments confirmed that UBD knockdown in PLFs significantly increased the expression levels of *Cxcl2* and *Cxcl3* and promoted neutrophil migration, supporting a regulatory role of UBD in shaping the fibrotic immune microenvironment. Consistent with our findings, Galati et al. reported reduced NK cells in IPF patients [37]. Although neutrophils are often elevated in the peripheral blood and bronchoalveolar lavage fluid of IPF patients and linked to poor prognosis [38, 39], we observed reduced neutrophil infiltration in fibrotic lung tissues, suggesting context-dependent effects. Neutrophil elastase is known to enhance fibrosis by promoting fibroblast activation and myofibroblast differentiation [40, 41]. However, some animal studies show that neutrophil depletion does not consistently mitigate fibrosis [42], underscoring the controversial role of neutrophils in IPF pathogenesis. These insights highlight the complex interplay between ubiquitination and immune regulation, which may inform future immunomodulatory therapies.

This study has several limitations. First, the expression of hub URGs requires validation in clinical IPF samples. Second, although UBD was shown to mediate TGFβ1-induced fibroblast activation, its crosstalk with neutrophils remains unclear. Third, the specific roles of the remaining five hub URGs in IPF pathogenesis warrant further investigation.

## Conclusion

In summary, our study delineated the ubiquitination-associated molecular landscape of IPF and established a robust six-gene diagnostic model with strong predictive performance. We further characterized immune dysregulation in IPF and identified significant correlations between hub URGs and immune cell infiltration. Experimental validation confirmed the expression patterns of these URGs and identified UBD as a functional effector of fibroblast activation. Collectively, our findings highlight the interplay between ubiquitin signaling and immune responses in IPF, offering novel insights and potential targets for therapeutic intervention.

## Supplementary Information

Supplementary file 1.

Supplementary file 2.

Supplementary file 3.

## Data Availability

The dataset analyses in this study were based on publicly available databases. Experimental data generated during the study are available from the corresponding author upon reasonable request.

## Abbreviations

IPF: Idiopathic Pulmonary Fibrosis
ECM: Extracellular Matrix
URGs: Ubiquitination-Related Genes
DEGs: Differentially Expressed Genes
DEURGs: Differentially Expressed Ubiquitination-Related Genes
GO: Gene Ontology
BP: Biological Processes
CC: Cellular Components
MF: Molecular Functions
KEGG: Kyoto Encyclopedia of Genes and Genomes
GSEA: Gene Set Enrichment Analysis
ssGSEA: Single-Sample Gene Set Enrichment Analysis
PCA: Principal Component Analysis
ROC: Receiver Operating Characteristic
AUC: Area Under the Curve
PLFs: Primary Lung Fibroblasts
TGFβ1: Transforming Growth Factor Beta 1
BLM: Bleomycin
H&E: Hematoxylin and Eosin
tSNE: t-distributed Stochastic Neighbor Embedding
LASSO: Least Absolute Shrinkage and Selection Operator
