# Supplementary material for: Decoding the ubiquitination-immunity axis in idiopathic pulmonary fibrosis: diagnostic insights and therapeutic implications
Source: Respir Res. 2026 Mar 6;27:170. doi: 10.1186/s12931-026-03612-7 (PMC13085684; doi:10.1186/s12931-026-03612-7)
Supplement: Supplementary file 2 [file 12931_2026_3612_MOESM2_ESM.docx]

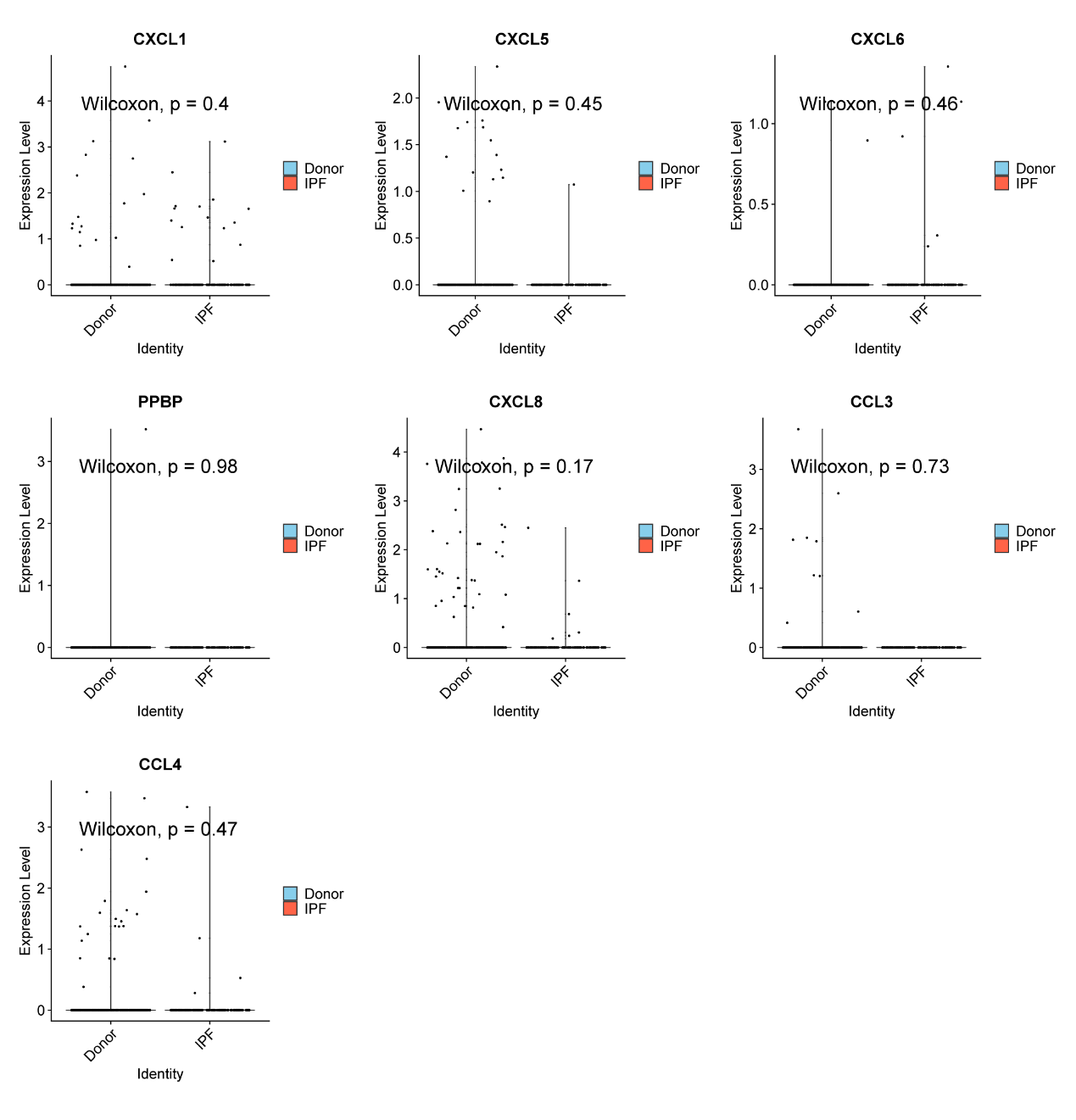


**Figure S1. Differential expression of *CXCL1*, *CXCL5*, *CXCL6*, *PPBP* (the coding gene of CXCL7), *CXCL8*, *CCL3*, and *CCL4* in fibroblasts from human IPF and control lungs in the scRNA-seq dataset GSE122960.**
