# Supplementary figures and images for: Decoding the ubiquitination-immunity axis in idiopathic pulmonary fibrosis: diagnostic insights and therapeutic implications
Source: Respir Res. 2026 Mar 6;27:170. doi: 10.1186/s12931-026-03612-7 (PMC13085684; doi:10.1186/s12931-026-03612-7)

**Figure 6H**

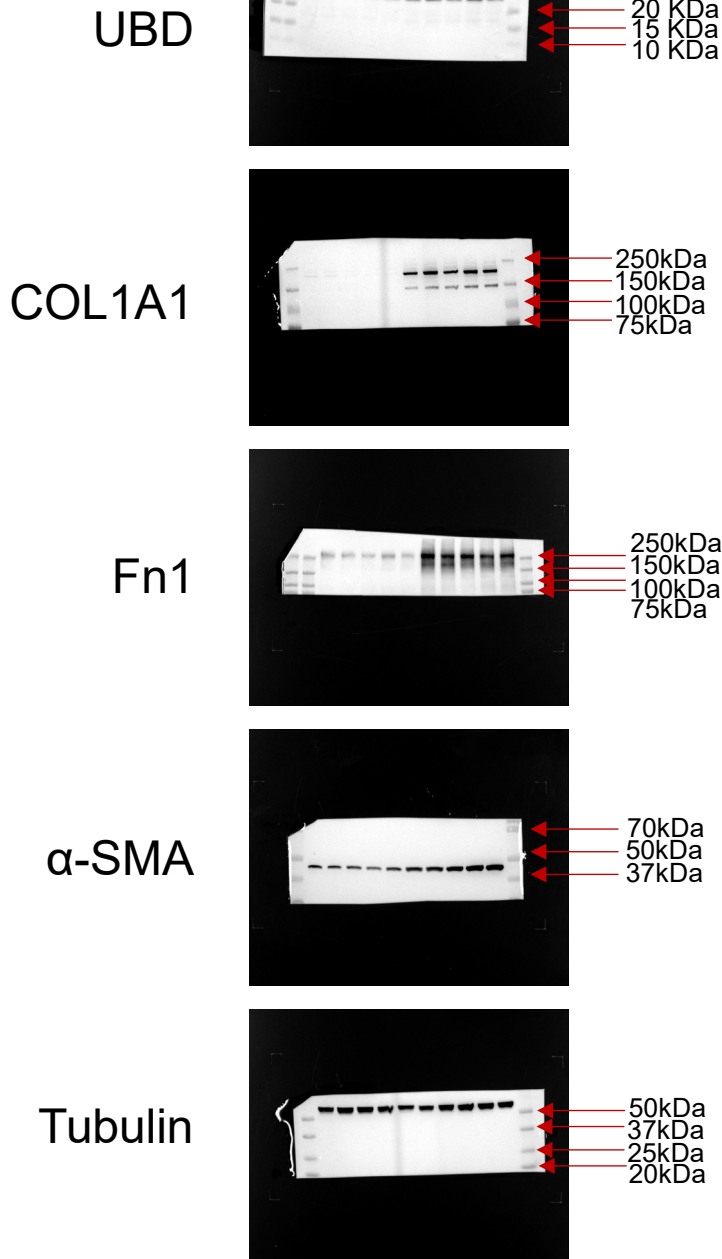

**Figure 6I**

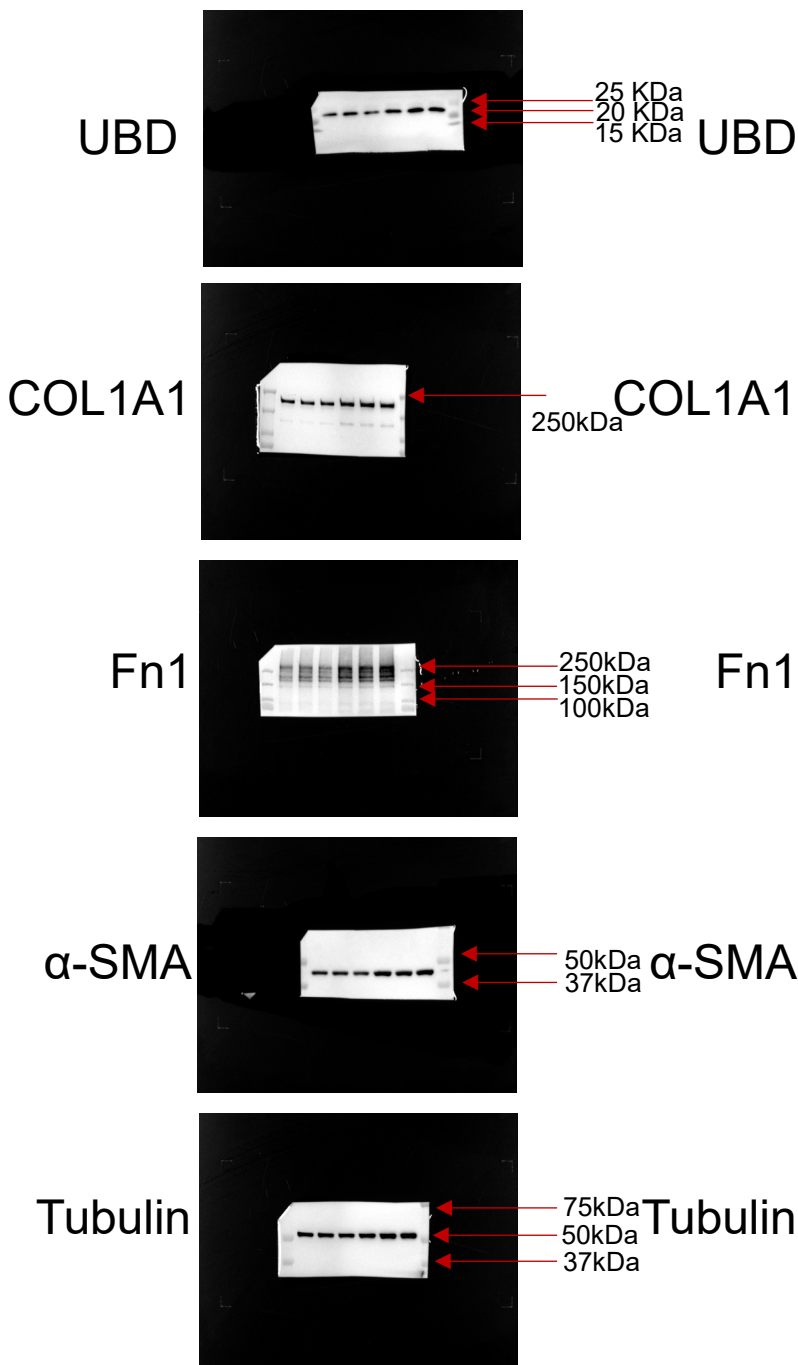

**Figure 6J**

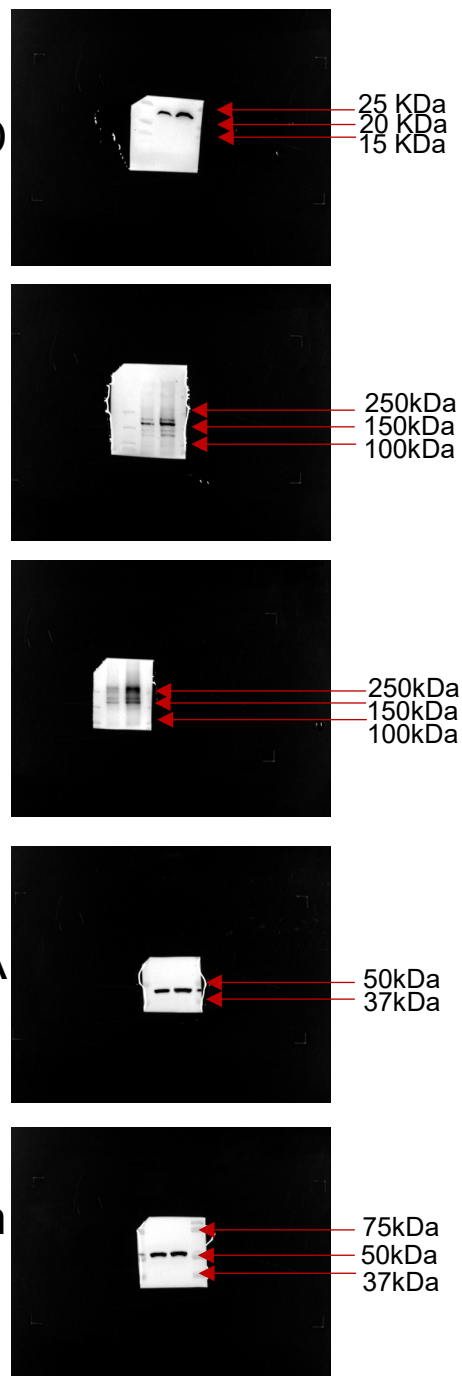

**Figure 7A**

UBD

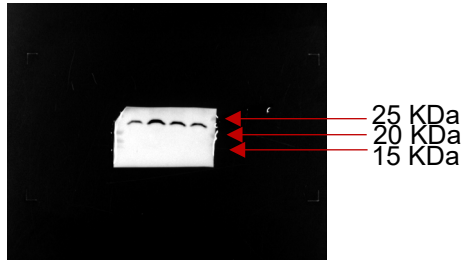

COL1A1

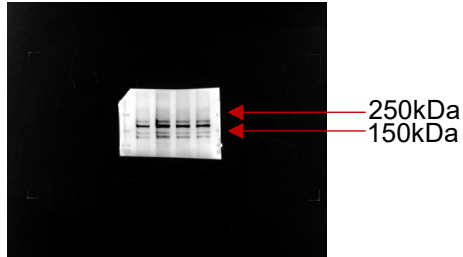

Fn1

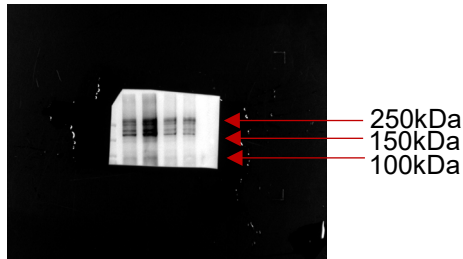

$\alpha$ -SMA

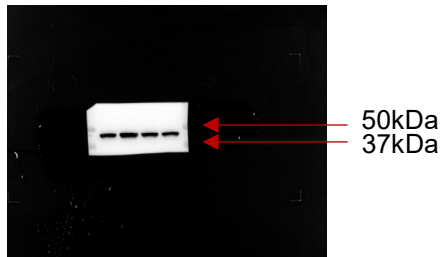

Tubulin

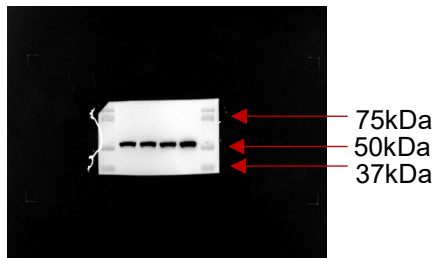

Supplement: Supplementary file 3 [file 12931_2026_3612_MOESM3_ESM.pdf]
